# Effect of Mirabegron in Men With Overactive Bladder and Erectile Dysfunction: A Prospective Observational Study

**DOI:** 10.7759/cureus.58175

**Published:** 2024-04-13

**Authors:** Rana P Singh, Arshad Jamal

**Affiliations:** 1 Department of Urology, Rajendra Institute of Medical Sciences, Ranchi, IND

**Keywords:** mirabegron, urine output, voiding, penile dysfunction, β3 adrenergic receptors

## Abstract

Background: As it has been observed that the erect penis has been the epitome of virility for the male community for decades, it became necessary to search for alternative treatments for the cause. So, the study was performed to evaluate the potential impact of mirabegron in men with mild to moderate erectile dysfunction (ED) and overactive bladder (OAB).

Methods:It was a prospective, observational study that was carried out at the Department of Urology at Rajendra Institute of Medical Sciences, Ranchi, for a duration of two years and included a total of two hundred fifty patients. The individuals included had a diagnosis of mild to moderate erectile dysfunction (ED) along with symptoms of OAB. The overactive bladder questionnaire (OAB-q) score and the International Index of Erectile Dysfunction-5 (IIEF-5) score were used, respectively, to measure the impact of mirabegron on ED and OAB. Then, the changes in ED and OAB were evaluated at two, four, eight, and 12 weeks.

Results: Among the total 250 patients recruited, around 32.5% of them had mild ED, 17.5% were diagnosed with mild to moderate ED, and 50% suffered from moderate ED. The IIEF-5 scores improved by four points or more in 86.25%, 91.25%, and 71.25% of patients after four, eight, and 12 weeks, respectively. OAB-q scores were likewise shown to decline in the fourth (13.1 ± 4.3) and eighth (12.8 ± 4.2) weeks when compared to the baseline (17.4 ± 5.5). Also, adverse events reported did not hamper the progress of the study.

Conclusion:The study concluded that mirabegron has a beneficial impact on controlling OAB symptoms among men diagnosed with mild to moderate ED. The effects last for only eight weeks, and then they decline. Furthermore, mirabegron was well-tolerated among patients and had no safety concerns with its use.

## Introduction

The term "erectile dysfunction" (ED), formerly known as "impotence," refers to the inability to attain or sustain the stiff penile erection required for satisfying sexual activity. The erect penis has been an epitome of virility for the male community for decades [1]. Phosphodiesterase-5 (PDE5) inhibitors, since their discovery, have been seen to revolutionize the management of ED [2]. Sildenafil, tadalafil, vardenafil, and avanafil are some of the known PDE5 inhibitors in clinical use. These molecules act by competitively inhibiting the PDE5 enzyme, enhancing the buildup of cyclic guanosine monophosphate (cGMP), with the subsequent release of a potent vasodilator, nitric oxide (NO). Due to their better safety and clinical efficacy, PDE5 inhibitors are frequently suggested as the first-line treatment of choice by clinicians [2,3]. The flaccidity of the smooth cavernosal muscle regulates penile erection, and upon release of NO, the cavernosal muscle relaxes and a successful erection is achieved [4]. It was recently discovered that PDE5 inhibitors had an overall effectiveness rate of up to 76% in treating ED [5]. Therefore, it is important to search for alternatives that target other pathways for those for whom PDE5 is contraindicated or unsuccessful.

One of the alternatives, the β3-receptors on activation in the human corpus cavernosum, causes cAMP-dependent vasorelaxation via the Rho-kinase pathway that is independent of NO release [6]. Other underlying mechanisms that facilitate penile erection include activation of the hydrogen sulfide pathway, a reduction in cytosolic calcium concentration, and activation of voltage-gated potassium channels, in addition to cGMP buildup mediated by NO release [7,8].

One of the β3 adrenergic receptor agonists, namely, mirabegron, is used for the treatment of troublesome overactive bladder (OAB) [9]. It accelerates the release of cAMP levels in the human urothelial cells, and this, in turn, promotes detrusor smooth muscle relaxation [10]. It also suppresses frequent muscle contractions by releasing urothelial-derived inhibiting factor (UDIF) [11]. Thus, it helps to alleviate the symptoms of lower urinary tract symptoms (LUTS), such as urge urine incontinence, urgency, and increased urinary frequency [12]. In previous studies, it has been observed that the diagnosis of ED was more frequently seen in men with LUTS than in men without LUTS [13,14]. A study performed in vivo using rats as an animal model for ED has demonstrated other mechanisms such as alpha-1 (α1) adrenoreceptor blockade in addition to cAMP accumulation [15]. However, the scientific literature does not completely support the use of mirabegron for ED among men with OAB, and there are conflicting findings about it [16].

Thus, the authors have explored the impact of using 25 mg of mirabegron (with successive increases if tolerated) on improvements in the International Index of Erectile Dysfunction-5 (IIEF-5) score for ED and the OAB-q score for OAB. Therefore, the aim of the study was to evaluate the outcome of mirabegron use among men with mild to moderate ED with OAB.

## Materials and methods

Study design

It was a prospective, observational study. The duration of the study was two years, carried out from November 2020 to December 2022. A total of 250 patients were selected who satisfied the inclusion criteria from the urology outpatient department at Rajendra Institute of Medical Sciences, Ranchi.

Study population

The inclusion criteria of participants were patients with the diagnosis of mild cases to moderate cases of ED (11 to 25 IIEF-5 score), patients with an age group of 18 to 70 years, OAB symptoms that for more than or equal to three months (at least eight micturition per month, at least three episodes of urinary urgency per day) [17]. The study excluded participants who were taking PDE5 inhibitors or other ED medications and had a history of priapism, prostatectomy, cystectomy, transurethral operations, or penile surgery. Additionally, participants with diseases of the central nervous system, Parkinson's disease, excessive blood pressure, chronic kidney disease, moderate to severe hepatic impairment, infection of the urinary tract, and a post-void residual amount (PVR) of >150 ml were all excluded from this study.

Ethical approval

The study has complied with the Declaration of Helsinki and has received informed consent from all the participants. Ethical approval has been granted by the Institutional Ethics Committee of Rajendra Institute of Medical Sciences, Ranchi (Memo No. 81 IAEC/IEC RIMS, Ranchi).

Data collection

All patients who opted to take part in the research study gave their informed consent. Patients recruited for the study were evaluated at baseline for the following-clinical history, blood pressure, PVR amount, urinalysis, the culture of urine samples, and evaluation of the ED using IIEF-5 scores [17] and OAB using the OAB-q questionnaire [18]. Patients were enrolled for a period of 12 weeks, where they received mirabegron 25 mg, which was administered for a period of 14 days. If tolerated, they could proceed with 25 mg or 50 mg for the next 10 weeks. Patients were monitored at two, four, eight, and 12 weeks, and at each visit, the participants were requested to repeat the IIEF-5 and OAB-q questionnaire, along with PVR measurement, blood pressure, urinalysis, and urine culture, and any adverse events (AEs) reported during medication administration.

The improvement in the overall mean IIEF-5 score of four or more and the ten-point decrease in the OAB-q symptom severity score from baseline were the study's main outcomes. The pre-treatment scores were compared with the scores obtained at two, four, eight, and 12 weeks.

Statistical analysis

Based on previous literature, we calculated the sample size using the formula n1 = (σ2 + σ2/K) (Z1-α/2 + Z1-β)/d2, at 95% power and 5% type-2 error; n2 = K*n1, d = difference between 2 means, σ2 = variance, k = ratio of sample size for groups 1 and 2, α = probability of type-1 errors, β = probability of type-2 errors, and Z = critical value given for α and β. So, a total of 250 patients were to be enrolled in the study.

Data contained both continuous and categorical variables. Quantitative variables were expressed as mean ± standard deviation (SD) or n (%) or n.

## Results

A total of 250 patients were recruited in this observational study. Out of them, 50 were lost to follow-up, and thus, only 200 patients were evaluated further. Table 1 shows the baseline and demographic information for these patients. Personal details such as age groups, related comorbidities, smoking, and alcohol history were also evaluated. Other than this, various parameters, such as categories of LUTS, ED etiology, and ED severity, were also assessed.

**Table 1:** Baseline and demographic characteristics. ED: erectile dysfunction, BMI: body mass index, LUTS: lower urinary tract symptoms, SD: standard deviation.

Parameters	Values
Number of patients	200
Mean patient age in years (SD)	60.2±6.78
Age groups (%)	
Less than 65	128 (64)
Older than 65	72 (36)
Mean BMI kg/m^2^ (SD)	30.1±6.65
Number of comorbidities present (%)	
Diabetes mellitus	49 (24.5)
Hypertension	37 (18.5)
Hyperlipidemia	29 (14.5)
Cardiovascular disease	17 (8.5)
Cerebrovascular accident	4 (2)
Smoking history (%)	117 (58.5)
Current alcohol use (%)	78 (39)
Categories of LUTS (%)	
Storage symptoms	148 (74)
Voiding symptoms	29 (14.5)
Mixed symptoms	4 (2.0)
Previous medication uses for LUTS (%)	105 (52.5)
Type of ED etiology (%)	
Vascular	49 (61.25)
Diabetic	17 (21.25)
Neurogenic	3 (3.75)
Others	11 (13.75)
ED severity (%)	
Mild	26 (32.5)
Mild to moderate	14 (17.5)
Moderate	40 (50)
Time since ED was diagnosed (%)	
Less than one year	49 (24.5)
Equal to or more than one year	142 (71)
Previous medication uses for ED (%)	54 (27)

**Figure 1:**
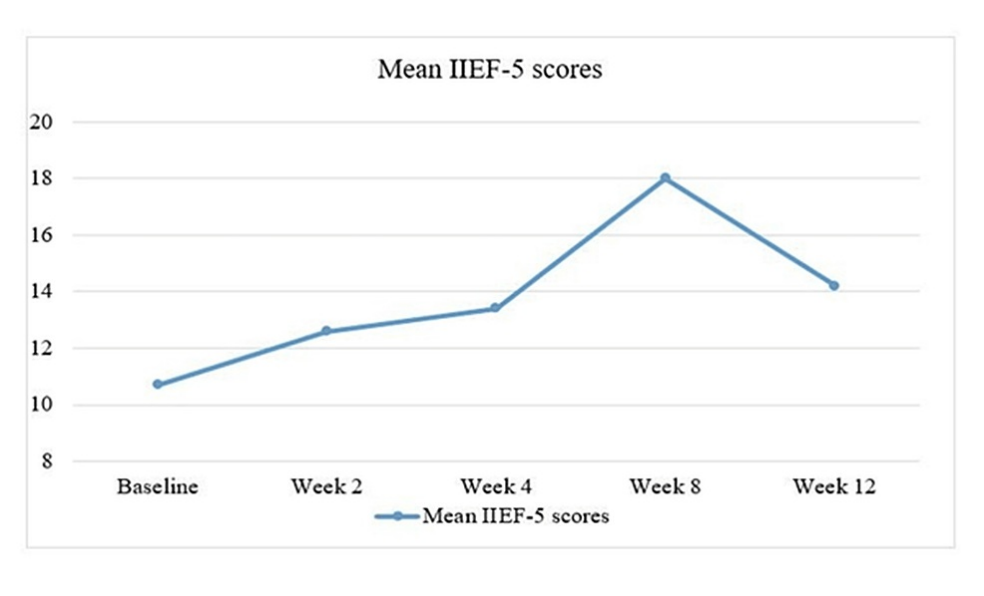
Mean IIEF-5 scores at two, four, eight, and 12 weeks after starting mirabegron compared to pre-treatment levels. IIEF-5: International Index of Erectile Dysfunction-5.

After 12 weeks, 142 (71%) individuals had shown an improvement of four points or greater in their corresponding IIEF-5 scores. However, mirabegron treatment did not affect IIEF-5 scores in 58 (29%) patients at 12 weeks. At week, a higher number of patients had an improvement of four or more points in the IIEF-5 scores, that is, 69 (34.5%) and 73(36.5%), respectively. Additionally, there were no clinically sound reductions in IIEF-5 scores. This suggests that mirabegron has a favorable effect on sexual dysfunction for up to eight weeks before it starts to diminish. Figure 1 depicts the improvement in the mean IIEF-5 score from baseline to two, four, eight, and 12 weeks after administration of mirabegron. The scores were found to be the highest at week eight and declined further.

Additionally, it was discovered that erectile function substantially improved throughout the 12-week study period; however, orgasmic ability, sexual stimulation, and intercourse contentment were not significantly impacted by mirabegron. Table 2 lists the change to the mean IIEF-5 scores for each parameter. In contrast to the baseline scores, it was also discovered that the OAB-q scores decreased more rapidly in the fourth and eighth weeks, as shown in Table 2. Similarly, PVR quantity decreased after the eighth week of treatment but was not significantly impacted by Mirabegron administration.

**Table 2:** IIEF-5, OAB-q scores, and PVR amount for each parameter. Data are presented as mean±SD. PVR: post-void residue, OAB: overactive bladder, IIEF-5: International Index of Erectile Dysfunction-5.

	Baseline	Week 2	Week 4	Week 8	Week 12
IIEF-5	10.7 ± 3.8	12.6 ± 3.8	13.4 ± 3.4	18 ± 5.2	14.2 ± 4.7
Erectile function	13.3 ± 5.6	14.7 ± 5.3	16.3 ± 4.6	18.7 ± 7.9	16.7 ± 6.8
Orgasmic ability	7.7 ± 1.9	6.8 ± 2.5	8.2 ± 2.2	8.6 ± 2.1	8.7 ± 2.4
Sexual stimulation	6.1 ± 1.8	5.4 ± 2.4	5.5 ± 1.8	6.2 ± 2.3	5.8 ± 2.2
Intercourse contentment	7.6 ± 3.2	6.8 ± 3.0	7.2 ± 3.7	9.3 ± 4.3	8.8 ± 4.7
Overall satisfaction	6.9 ± 2.0	6.5 ± 2.2	7.1 ± 2.3	6.7 ± 2.8	6.3 ± 2.4
OAB-q scores	18.4 ± 5.5	16.6 ± 5.5	14.1 ± 4.3	13.8 ± 4.2	13.7 ± 4.3
PVR amount (ml)	38.5 ± 43.8	43.8 ± 34.9	36.9 ± 39.4	31.8 ± 29.8	55.1 ± 68.4

AEs reported during mirabegron treatment were rare and similar to the previously reported side effects of mirabegron. A list of adverse effects of the drug mirabegron seen during the study period were headache, elevated blood pressure, nasal congestion, urinary tract infection, constipation, leg pain, back pain, gastric discomfort, flu-like symptoms, and rash. Although, no serious adverse effects were reported during the study.

## Discussion

This study assessed the effect of mirabegron on mild, mild to moderate, or moderate ED in male OAB patients. Almost 71.25% of the patients had clinically improvement in ED, which was observed by an improvement in the mean score of four points or more in the IIEF-5 questionnaire over a period of 12 weeks. However, the study has not shed light on the long-term utility of mirabegron for the treatment of ED and has not evaluated its efficacy among men with severe ED. Also, the increased mean IIEF-5 score was found to decline slowly from the 8th week onwards. Furthermore, mirabegron also improved OAB without affecting the amount of PVR.

The primary objective of this research was to analyze the effect of mirabegron on improving the IIEF-5 scores among men with mild to moderate ED. The IIEF-5 score was selected due to its convenient patient reporting and also due to its simple-to-comprehend nature [19]. A study done by Rosen et al. predicted that an improvement in the IIEF-5 score by four points or greater is deemed to be clinically meaningful, with an estimated sensitivity and specificity of 0.74 and 0.73, respectively [20]. Understanding therapeutic efficacy requires placing mirabegron-related changes in the context of clinically relevant scores. However, depending on the type of ED being considered, these clinically meaningful scores can go as low as two for mild ED [20].

The effect of mirabegron treatment has also been assessed by the authors on the other IIEF-5 indicators, which include orgasmic ability, sexual stimulation, intercourse contentment, and overall satisfaction. By the end of the eighth week, an improvement in each of the aforementioned indicators was noted, which corresponds to the improvement in erectile function. In line with the findings from a randomized trial conducted by Althof et al., sexual stimulation was found to be higher among those with less severe ED [21].

OAB may be treated with mirabegron, a selective agonist of the β3 adrenergic receptor. It was discovered that taking mirabegron orally for 12 weeks improved incontinence, urgency, and frequency of urination. PVR was unaltered throughout the study. The authors advise long-term studies to determine mirabegron's effectiveness and tolerability in treating OAB, particularly in those for whom antimuscarinics are inappropriate or contraindicated [22]. Chapple et al. [23] have also recommended that a daily dose of 25 mg, 50 mg, or 100 mg demonstrates clinically relevant benefits among those with OAB. Our findings are consistent with those from earlier research, which were made evident by the OAB-q questionnaire given to study participants at various points. Mirabegron was also found to be well-tolerated during the study with expected AEs. The most commonly reported AEs so far are increased blood pressure, nasal congestion, urinary tract infection, headache, constipation, abdominal pain, and back pain among others [24]. However, the AEs in this study were found to be transient and short-lived, with less severity. As evidenced in previous studies, mirabegron has a beneficial impact on men suffering from ED and benign prostatic hyperplasia [25]. However, a clear understating of its long-term efficacy and AE profile is essential to advocate its use in clinical settings. Being an alternative to anti-muscarinic therapy for OAB, its utility in ED management remains to be explored through long-term studies.

The study had several limitations, such as the study was single-centric, small sample size, and a brief study period. Other than this, the authors were unable to explain the diminishing effects of mirabegron's after eight weeks. Initially, the authors included people who have been taking medications for erectile dysfunction; however, they were unable to incorporate the washout period, which is another limitation of this study. Apart from the washout period, data of the exact medications being employed for lower urinary tract symptoms were not collected in this study. So, future studies are needed to determine how mirabegron affects other cohorts, particularly individuals with chronic conditions, and assess the impact of various mirabegron doses on erectile function in males with ED.

## Conclusions

It is concluded that mirabegron treatment has a beneficial role in controlling OAB among men diagnosed with mild to moderate ED. The study also concludes that the effect of treatment with mirabegron lasts for up to eight weeks and then declines. Additionally, it should be noted that taking mirabegron may not increase the volume of post-void residue. Scores of the questionnaire IIEF-5 were also not increased in participants after treatment with mirabegron, and simultaneously, OAB-q scores decreased in the fourth and eighth week. Although, it has been observed that mirabegron was well-tolerated, and no serious safety concerns were raised in terms of urinary retention or cardiovascular events. Further research may help to assess the role of mirabegron in chronic conditions.
